# Pathoadaptive Mutations of *Escherichia coli* K1 in Experimental Neonatal Systemic Infection

**DOI:** 10.1371/journal.pone.0166793

**Published:** 2016-11-18

**Authors:** Alex J. McCarthy, David Negus, Patricia Martin, Catarina Pechincha, Eric Oswald, Richard A. Stabler, Peter W. Taylor

**Affiliations:** 1 University College London School of Pharmacy, London, United Kingdom; 2 UMR1220 Inserm, Toulouse, France; 3 UMR 1416 INRA, Toulouse, France; 4 Université de Toulouse, UPS, Institut de Recherche en Santé Digestive, Toulouse, France; 5 CHU Toulouse, Hôpital Purpan, Service de Bactériologie-Hygiène, Toulouse, France; 6 London School of Hygiene and Tropical Medicine, London, United Kingdom; Animal and Plant Health Agency, UNITED KINGDOM

## Abstract

Although *Escherichia coli* K1 strains are benign commensals in adults, their acquisition at birth by the newborn may result in life-threatening systemic infections, most commonly sepsis and meningitis. Key features of these infections, including stable gastrointestinal (GI) colonization and age-dependent invasion of the bloodstream, can be replicated in the neonatal rat. We previously increased the capacity of a septicemia isolate of *E*. *coli* K1 to elicit systemic infection following colonization of the small intestine by serial passage through two-day-old (P2) rat pups. The passaged strain, A192PP (belonging to sequence type 95), induces lethal infection in all pups fed 2–6 x 10^6^ CFU. Here we use whole-genome sequencing to identify mutations responsible for the threefold increase in lethality between the initial clinical isolate and the passaged derivative. Only four single nucleotide polymorphisms (SNPs), in genes (*gloB*, *yjgV*, *tdcE*) or promoters (*thrA*) involved in metabolic functions, were found: no changes were detected in genes encoding virulence determinants associated with the invasive potential of *E*. *coli* K1. The passaged strain differed in carbon source utilization in comparison to the clinical isolate, most notably its inability to metabolize glucose for growth. Deletion of each of the four genes from the *E*. *coli* A192PP chromosome altered the proteome, reduced the number of colonizing bacteria in the small intestine and increased the number of P2 survivors. This work indicates that changes in metabolic potential lead to increased colonization of the neonatal GI tract, increasing the potential for translocation across the GI epithelium into the systemic circulation.

## Introduction

*Escherichia coli* is a major causative agent of neonatal septicemia, sepsis, and meningitis [1,2] with the majority of isolates expressing the K1 capsular antigen [3,4], a linear homopolymer of α-2,8-linked *N*-acetylneuraminic acid [5] that is subject to varying degrees of O-acetylation [6]. Predisposition to *E*. *coli* K1 neonatal infection results from vertical transmission of the bacteria from mother to infant at or soon after birth [7]. Strains may colonize the neonatal gastrointestinal (GI) tract and in some cases translocate from the intestinal lumen into the systemic circulation, enter the central nervous system and gain access to the meninges [7–9]. *E*. *coli* K1 systemic infections are characterized by a strong age-dependency: the bacteria are benign, common constituents of the adult gut microbiota [9] but are potentially lethal during the first four weeks of life [2,10].

The molecular mechanisms of *E*. *coli* K1 pathogenesis in neonatal infection have proven difficult to unravel. Fortunately, many of the features of human infections can be replicated in the neonatal rat and this model has been instrumental in defining the key events that determine invasive disease. A stable GI-colonizing population can be established by feeding a bolus of *E*. *coli* K1 cells to neonatal rat pups [11,12]. Pups colonized at two days of age (P2) are exquisitely susceptible to systemic infection but become progressively more resistant as the animals mature [12,13], and after one week (P9) the bacteria are unable to invade the blood circulation from the gut lumen [13,14] due to maturation of the protective mucus layer of the small intestine over the P2-P9 period [13]. *E*. *coli* K1 isolates differ markedly in their capacity to colonize the GI tract and to traverse from the gut to the systemic circulation via mesenteric lymph [11,12]. For example, Achtman and colleagues [12,15] examined the capacity of 95 *E*. *coli* K1 isolates to elicit bacteremia after colonization of the GI tract; colonization was uniformly high with rates of 88–100% but there was large strain-to-strain variation in rates of bacteremia (2–50%) that were highly correlated with O-serotype; lethality also varied significantly between isolates, ranging from 0–25%.

We have utilized the neonatal rat to evaluate novel therapeutics and to examine in detail the pathogenesis of *E*. *coli* K1 systemic infection [16]. In order to establish a reproducible model with a high rate of colonization and systemic infection, we selected *E*. *coli* K1 A192 from the studies of Achtman and coworkers; this septicemia isolate [15] was reported to have colonized all 64 newborn rats tested with an incidence of bacteremia of 35% and survival of 75% [12]. We were able to enhance the virulence of *E*. *coli* A192 by two rounds of passage through susceptible P2 neonatal rats to obtain a derivative, *E*. *coli* A192PP, that produced lethal infections of the blood circulation and major organs, including those of the central nervous system, in all P2 animals [17,18]. In this report we characterize the mutations responsible for the enhanced virulence of *E*. *coli* A192PP and the intermediate blood isolate A192P.

## Materials and Methods

### Bacterial strains

*E*. *coli* A192 (O18:K1) was isolated in 1977 from the blood of a Dutch patient with septicemia [15] and was obtained from Deutsche Sammlung von Mikroorganismen und Zellkulturen (DSMZ), Braunschweig, Germany as DSMZ 10719. *E*. *coli* derivatives A192P and A192PP were obtained by serial passage in P2 neonatal rats; a colony isolated from blood culture of an animal colonized by *E*. *coli* A192 was designated A192P, and a colony isolated from blood culture of an animal colonized by A192P was designated A192PP [19]. Inactivation of *E*. *coli* A192PP genes was undertaken using phage λ Red recombinase [20]. The primers used to construct mutants are shown in Table 1. Allelic exchanges were confirmed by PCR. For *in vitro* growth rate studies, M9 medium was supplemented with various carbon sources at a concentration of 0.2%; growth was monitored every hour over a 15 h period.

**Table 1:** Primers used in this study for mutagenesis of *E*. *coli* A192PP.

Primer	Sequence 5’ to 3’
*gloB*-P1	TGAGAGGTAATCTATGAATCTTAACAGTATTCCCGCCTTTGATGACAATTGTGTAGGCTGGAGCTCTTC
*gloB*-P2	GACAAGAAAGTTTTATCAGAACCTATCTTTCTTTGACCTTAACCATGCAACCATATGAATATCCTCCTTAG
*yjgV*-P1	GCCCAGGGCGTGGGTGTCATCGCCTTTCTGATTGGTATCACAACATTTTTGTGTAGGCTGGAGCTCTTC
*yjgV*-P2	AAGGGGTTTTCTCCACTTTAAACGGATCAATTCCCCTTCTCTGCATACGCCATATGAATATCCTCCTTAG
*tdcE*-P1	GGATGCGCCATTAAAGCGTGCGCTGCATCCGTTCGGCGGCATTAATATGAGTGTAGGCTGGAGCTCTTC
*tdcE*-P2	CTTCGACATCCGCTTCGTGGTGGAAATACCCATCCAGCAGGCCGACAAGGCATATGAATATCCTCCTTAG
*thrA*-P1	GGTACATCAGTGGCAAATGCAGAACGTTTTCTGCGGGTTGCCGATATTCTGTGTAGGCTGGAGCTCTTC
*thrA*-P2	ACAGATTCGCCATAAAAGCGGCGACATCACCCTCGGCGTTAAACTCTGCGGCATATGAATATCCTCCTTAG

### Genome sequencing

The *E*. *coli* A192PP genome was sequenced for a previous study [21]. *E*. *coli* A192 and A192P were cultured overnight in Mueller-Hinton (MH) broth at 37°C using an orbital incubator (200 orbits/min). Genomic DNA was extracted from 1ml of culture using a QIAamp DNA minikit (Qiagen, UK). Library preparation for sequencing was performed with the Illumina Nextera XT kit according to manufacturer’s instructions. MiSeq (Illumina) sequencing was carried out using the MiSeq Reagent Kit v2 (2 x 151bp) following standard protocol. Sequencing reads were quality controlled using Trimmomatic [22]. To assemble the draft A192 genome, trimmed sequencing reads were assembled using Velvet [23] and VelvetOptimiser [24]. *x*BASE was used to predict protein coding sequences (CDS) and tRNA genes [25]. Functional annotation of CDS was performed by manual BLAST searches and genomic features identified with GIPSy software [26]. The *E*. *coli* A192 draft genome was compared with published *E*. *coli* genome sequences using Artemis Comparison Tool [27] and BLAST ring image generator [28]. To assess the evolutionary relationship between *E*. *coli* A192 and contemporary bloodstream isolates (BSI), trimmed fastq files were mapped against the *E*. *coli* IHE3034 genome using the Burrows-Wheeler Aligner software package [29]. SNPs were identified and filtered using samtools [30]. SNPs that fell within known phage sequences integrated into the IH3034 genome were removed from analysis. A phylogeny was constructed using FastTree [31] under default settings. To identify SNP mutations present in *E*. *coli* A192P and A192PP, trimmed fastq files were mapped against the A192 genome using the Burrows-Wheeler Aligner software package [29]. DELLY [32] was used to identify structural variants. Mapped reads were visualised on the A192 genome with Artemis [33].

### 2D SDS-PAGE

Cell proteomes of *E*. *coli* strains were analyzed by 2D gel electrophoresis using a Protean IEF Cell (Bio-Rad). Cells in mid-logarithmic phase (Luria broth; 37°C; 200 orbits/min) were collected by centrifugation, suspended in 1 ml of sample buffer (7 M urea, 2 M thiourea, 4% w/v CHAPS and Roche complete protease inhibitor), transferred to tubes containing Lysing Matrix B (MP Bio, Santa Ana, CA), and disrupted using a FastPrep FP120 cell homogenizer (ThermoFisher, Waltham, MA). Supernatants were recovered by centrifugation and proteins (300 μg) separated using 17 cm immobilized pH (range 3–10) gradient strips (Bio-Rad). Voltage was increased by gradient from 250 to 10,000 V with a final phase of 10,000 V for 60,000 V-h. SDS-PAGE was performed with 12% polyacrylamide gels (4°C, 60 mA) and proteins stained with Coomassie Brilliant Blue R-250. Gels were scanned using a Molecular Imager FX (Bio-Rad), and processed by PDQuest Advanced 2D-image analysis software (Bio-Rad). Significant changes in protein expression were identified by Students *t*-distribution (p<0.05); proteins were identified by LC-MS/MS after spot excision from the gel matrix.

### Colonization and infection of neonatal rats

Animal experiments were approved by the Ethical Committee of the UCL School of Pharmacy and the United Kingdom Home Office (HO) and were conducted in accordance with national legislation. Timed-birth Wistar rat pup litters (usually *n* = 12) were purchased from Harlan UK, delivered at P2 and colonized on the same day. Pups were retained throughout each experiment with the natural mothers in a single dedicated cage under optimal conditions (19–21°C, 45–55% humidity, 15–20 changes of air/h, 12 h light/dark cycle) and were returned to the mother immediately after colonization. Mothers had unrestricted access to standard rat chow and water. The procedure has been described in detail [16]. In brief, all members of P2 rat pup litters were fed 20μl of mid-logarithmic-phase *E*. *coli* (2–6 x 10^6^ CFU) from an Eppendorf micropipette. GI colonization was confirmed by culture of perianal swabs on MacConkey agar and bacteremia detected by MacConkey agar culture of blood taken post mortem. Disease progression was monitored by daily evaluation of symptoms of systemic infection and neonates culled by decapitation and recorded as dead once a threshold had been reached, as described earlier [16]: pups were regularly examined for skin color, agility, agitation after abdominal pressure, presence of a milk line, temperature, weight and behavior in relation to the mother. Neonates were culled immediately when abnormalities for three of these criteria were evident. After sacrifice, GI tissues were excised aseptically without washing, colon separated and the SI segmented into 2 cm portions representing proximal, middle and distal SI tissue. Tissues were then transferred to ice-cold phosphate-buffered saline, and homogenized. Bacteria were quantified by serial dilution culture on MacConkey agar supplemented with 25μg/ml kanamycin as appropriate. The presence of *E*. *coli* K1 was confirmed with phage K1E [34]: 20 lactose-fermenting colonies were streaked onto MH agar, 10 μl of ~10^9^ PFU/ml phage suspension dropped on each steak and the plates incubated overnight. *E*. *coli* K1 bacteria were quantified by multiplying total CFU by the proportion of K1E susceptible colonies. In all cases at least 19 colonies were susceptible to the K1 phage; *E*. *coli* K1 was never found in samples from non-colonized pups.

### Sequence data accession numbers

*E*. *coli* A192 and A192P raw sequence data and draft assembly of A192 genome have been deposited in the European Nucleotide Archive (project accession number PRJEB14305). A192PP has been previously deposited under PRJEB9141 [21]. Raw sequence data of the genomes of deletion mutants *E*. *coli* A192PPΔ*gloB*, A192PPΔ*yjgV*, A192PPΔ*tdcE* and A192PPΔ*thrA* generated during the current study are deposited as PRJEB16318.

## Results

### Serial passage of *E*. *coli* A192 in neonatal rats influences colonization of the GI tract and enhances virulence

**Fig 1:**
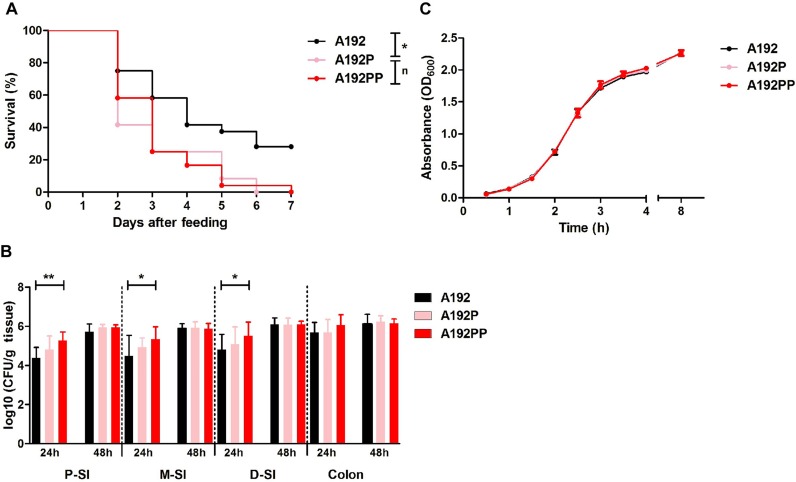
*E*. *coli* A192PP has enhanced capacity to colonize the GI tract and induce systemic infection in susceptible neonatal rats. (A) Survival of P2 rat pups fed 2–6 x 10^6^ *E*. *coli* K1 A192, A192P or A192PP (all, *n* = 24). Log-rank (Mantel-Cox) test; ns, nonsignificant; *, *P* < 0.05; **, *P* < 0.01). (B) Quantification of viable *E*. *coli* K1 in intestinal sections: tissues were removed 24 or 48 h after feeding, and CFU/g tissue determined by plating onto MacConkey agar. *E*. *coli* K1 colonies were identified with K1-specific phage. At 24 h, strain A192 *n* = 11, strain A192P *n* = 12, strain A192PP *n* = 12. At 48 hours, strain A192 *n* = 12, strain A192P *n* = 11, strain A192PP *n* = 11; Student's *t* test; *, *P* < 0.05; **, *P* < 0.01. (C) Growth of *E*. *coli* strains A192, A192P and A192PP in Mueller-Hinton (MH) broth at 37°C (200 orbits/min) (±SD, *n* = 3).

In a previous study [19], we found a similar incidence of bacteremia in neonatal rats colonized with *E*. *coli* A192 to that reported by Pluschke et al. [12,15], indicating that in the majority of animals the colonizing bacteria were unable to translocate from gut lumen to the systemic circulation and/or survive in the blood compartment. In contrast, all pups colonized with *E*. *coli* A192PP succumbed to bacteremia. Oral administration of the passaged strains *E*. *coli* A192P and A192PP to P2 rat pups produced a uniformly lethal infection, with all animals succumbing within 7 days (Fig 1A). To determine the capacity of the strains to colonize the GI tract of P2 rat pups, 2 cm segments from the proximal (PSI), mid- (MSI) and distal small intestine (DSI) as well as sections of the colon were excised following oral administration of *E*. *coli* A192, A192P and A192PP. The *E*. *coli* K1 content of each segment was then determined 24 and 48 h after colonization by viable counting (Fig 1B). *E*. *coli* K1 populations were established in the small intestine (SI) and colon within 24 h but the size of the colonizing A192PP cohort was significantly higher than A192 in all regions of the SI. These differences were not evident 48 h after oral administration of bacteria. No significant differences were found in the size of the colonizing pool in the colon at either time point. Thus, in quantitative terms, the passaged strains had a greater capacity to colonize the SI, the likely site of translocation from gut lumen to blood [13], than the wildtype strain A192; this trait was not linked to an enhanced growth rate in the nutrient-replete medium MH broth (Fig 1C).

### The A192 genome is comparable to other sequenced *E*. *coli* K1 genomes

*E*. *coli* A192 was selected for our investigations of K1 molecular pathogenesis in neonatal infections because of its attractive properties for establishment of rodent models of infection [16,17]. At the time, neonatal meningitis *E*. *coli* K1 isolates had been assigned to three groups, O1:K1, O7:K1 and O18:K1, on the basis of the seroreactivity and other phenotypic traits of six bacterial clones widespread in the pre-1981 period [15]. O7:K1 and O18:K1 isolates (the latter including *E*. *coli* A192) were considered more virulent than O1:K1 isolates as they elicited bacteremia in rodent models of neonatal infection and were able to proliferate in the bloodstream of these animals [12]. However, sequence-based molecular methodologies have profoundly increased understanding of the detailed population structures of extraintestinal pathogenic *E*. *coli* isolates [35,36]; we therefore evaluated the phylogenetic relationship of *E*. *coli* A192 to contemporaneous clinical strains by whole-genome sequencing.

**Fig 2:**
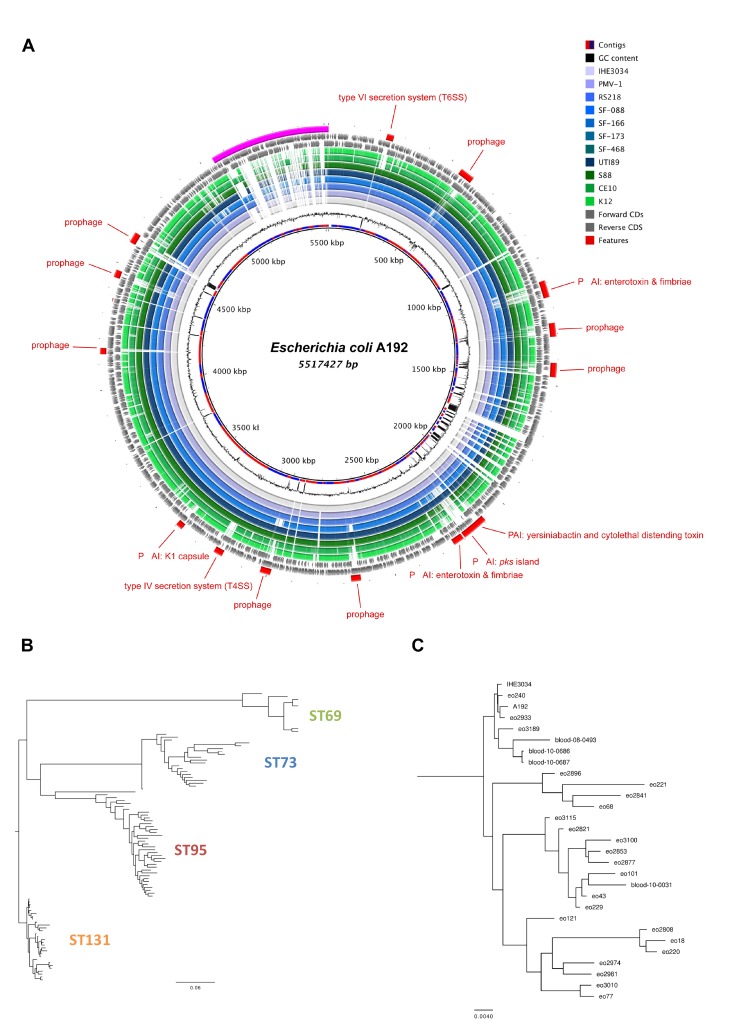
The *E*. *coli* A192 genome is comparable to other sequenced *E*. *coli* K1 genomes. (A) The *E*. *coli* K1 A192 genome was assembled and contiguous consensus sequences ordered according to the IHE3034 genome. From the inside, the circles represent contigs coloured in alternating red/blue, GC content, BLAST analysis of *E*. *coli* completed genomes IHE3034, PMV-1, RS218, SF-048, SF-166, SF-173, SF-468, UTI89, S88, CE10 and K12, forward CDS, reverse CDS, virulence factors and unmapped contigs. (B) Phylogenetic relationships of *E*. *coli* bloodstream infection isolates; strains were grouped into four sequence types: ST69 (green) ST73, (blue) ST95 (red) and ST131 (yellow). (C) Phylogenetic relationships of *E*. *coli* bloodstream infection ST95 isolates. *E*. *coli* A192 is closely related to reference genome IHE3034 and contemporary bloodstream infection isolates.

The *E*. *coli* A192 genome was sequenced to a depth of 100x and the draft genome assembled into 627 contiguous consensus sequence fragments consisting of 5,517,427 bps, with a 50.6% G+C content and predicted to contain 5,668 coding domain sequences. Multilocus sequence type data was extracted from the draft genome and *E*. *coli* A192 was assigned to sequence type (ST) 95. Pairwise comparisons of *E*. *coli* K1 genomes confirmed the presence in *E*. *coli* A192 of a stable collinear backbone and carriage of a number of characterised virulence factors (Fig 2A). To determine if *E*. *coli* A192 is representative of contemporary *E*. *coli* bloodstream isolates, we identified single nucleotide polymorphisms (SNPs) for 99 genomes against *E*. *coli* O18:K1 reference strain IHE3034 (accession number CP001969) and constructed a phylogenetic tree (Fig 2B). The *E*. *coli* A192 genome clustered within a clade of ST95 isolates, consisting of the reference strain IHE3034 (isolated in 1976 in Finland) and isolates eo240 (2001, UK), eo2933 (2010, UK), eo3189 (2010, UK), blood-08-0493 (2008, USA), blood-10-0686 (2010, USA) and blood-10-0687 (2010, USA). To identify genomic features of *E*. *coli* A192, we analysed the genome using genomic island prediction (GIPSy) software and identified 36 genomic islands that varied in size from 5,385 to 149,643 bps. Many of these harbored genes coding for known or putative virulence factors, including colibactin, enterobactin, enterotoxin, fimbrial proteins and secretion systems. The islands harbor genes coding for putative virulence factors, phage components and metabolic enzymes, such as type VI secretion systems (island I), lateral flagellar proteins (island II), lipopolysaccharide (island X), type III secretion systems (island XIII), polysialic acid capsular polysaccharide (island XIV), heme transporter systems (island XVI), invasins (island XVII), adhesins (island XXII), prophage (island V-VII, XI, XII, XIX) and a hydroxyphenylacetic acid- metabolizing enzyme. We identified 24 genomic islands greater than 9 kb in size that were absent in *E*. *coli* MG1655 and W3110 genomes.

### *E*. *coli* K1 A192PP has accumulated pathoadaptive mutations

**Fig 3:**
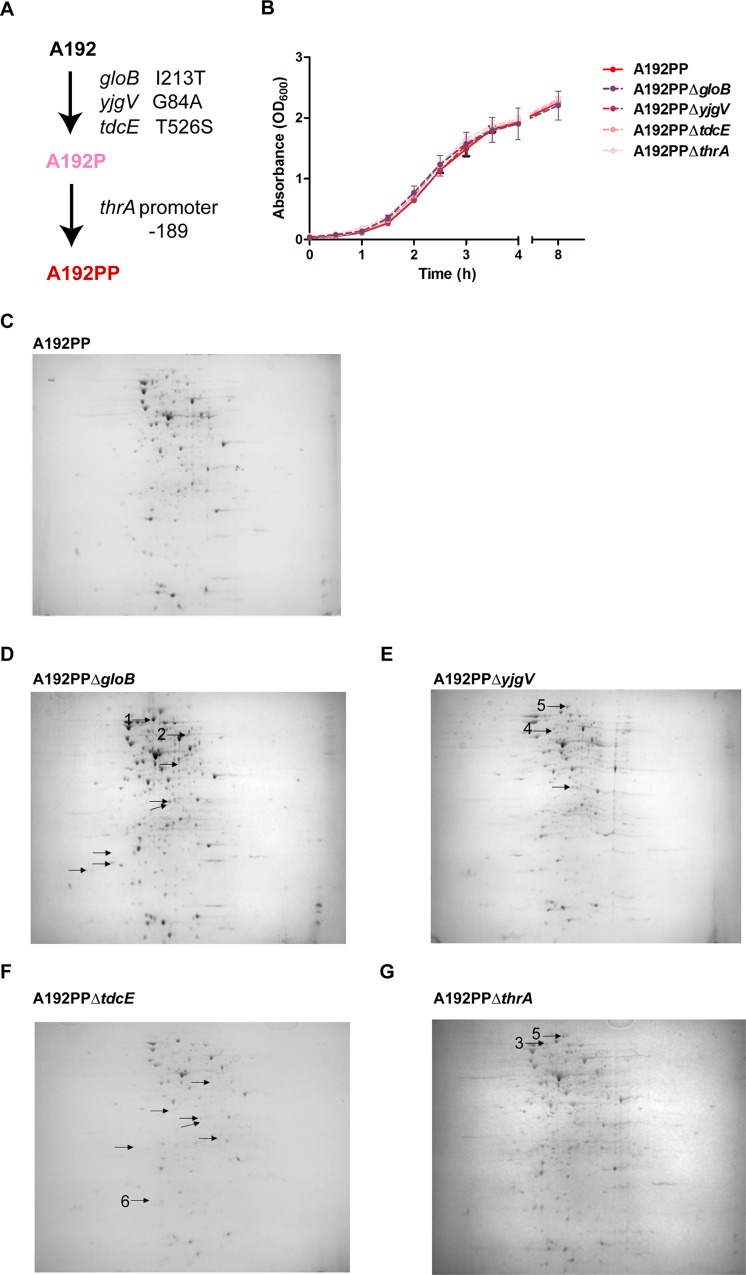
*E*. *coli* A192PP has acquired mutations that alter the proteome. (A) Genes acquiring single nucleotide polymorphisms during passage through infection-susceptible P2 rat pups. (B) Growth of *E*. *coli* A192PP strains in MH broth at 37°C (200 orbits/min) (±SD, *n* = 3). Total protein from (C) A192PP, (D) A192PPΔ*gloB*, (E) A192PPΔ*yjgV*, (F) A192PPΔ*tdcE* and (G) A192PPΔ*thrA* were separated on 2D gels using immobilized pH gradient strips in the range 3–10. The gels were stained with Coomassie Brilliant Blue, scanned and differences in protein abundance detected using PDQuest software. Proteins that differed at least twofold (*P*>0.5) are indicated. Proteins were identified by LC/MS/MS; numbered spots represent enoyl-acyl carrier protein reductase (1), transketolase (2), pyruvate dehydrogenase E1 component (3), ATP-dependent protease ATPase subunit (4), elongation factor G (5) and thiol peroxidase (6).

We previously determined that *E*. *coli* A192 elicited bacteremia in a minority (23%) of P2 rat pups following GI tract colonization of all littermates, in broad agreement with data (35%) from the study of Pluschke et al. [12]. Whole-genome sequencing of *E*. *coli* A192P and A192PP indicated that the initial passage, yielding *E*. *coli* A192P, resulted in the acquisition of one SNP in each of three genes, *gloB*, *yjgV* and *tdcE* (Fig 3A). *gloB* encodes a hydroxyacylglutathione hydrolase that catalyses the hydrolysis of S-D-lactoyl-glutathione to glutathione and D-lactic acid during methylglyoxal detoxification [37]. The SNP T638C, I213T has been described in *E*. *coli* (GenBank Accession: WP_052869873); it was not located in any characterized functional domain but at the surface of the predicted 3D structure (ProteinModalPortal: P0AC84, based on structure of the *S*. *enterica* Typhimirium molecule, with 78% homology). *yjgV* encodes a probable alcohol dehydrogenase [38,39]. The *yjgV* substitution (G251C, G88T) has also been described in *E*. *coli* (GenBank Accession: WP_052870045). The predicted 3D structures possess ≤26% homology, making it difficult to infer the functional role of residue 88. *tdcE* encodes a 2-keto acid formate-lyase involved in the anaerobic catabolism of L-threonine [40,41]. The *tdcE* substitution (A1576G, T526S) was not present in any protein sequence in the GenBank database. In similar fashion to the GloB substitution, amino acid 526 was not located in any annotated functional domains but at the surface of the predicted 3D structure (ProteinModalPortal: P42632, based on structure of the *E*. *coli* protein with 80% homology). The second passage produced an additional SNP in the promoter region of *thrA* (C→A at -189), controlling expression of the bifunctional protein aspartokinase I-homoserine dehydrogenase I that is involved in the conversion of L-aspartate to L-threonine, L-lysine, L-methionine and L-isoleucine [42,43]. We found no evidence for the acquisition or loss of genes encoded on the chromosome or carried on mobile genetic elements in the evolved strains, or for the existence of structural variants in either *E*. *coli* A192P or A192PP. However read mapping and assembly are limited with regard to identification of larger insertions, deletions, rearrangements, duplications and mobile genetic elements. The use of long-read sequencing such as PacBio or MinION would unambiguously clarify the existence of such events.

**Fig 4:**
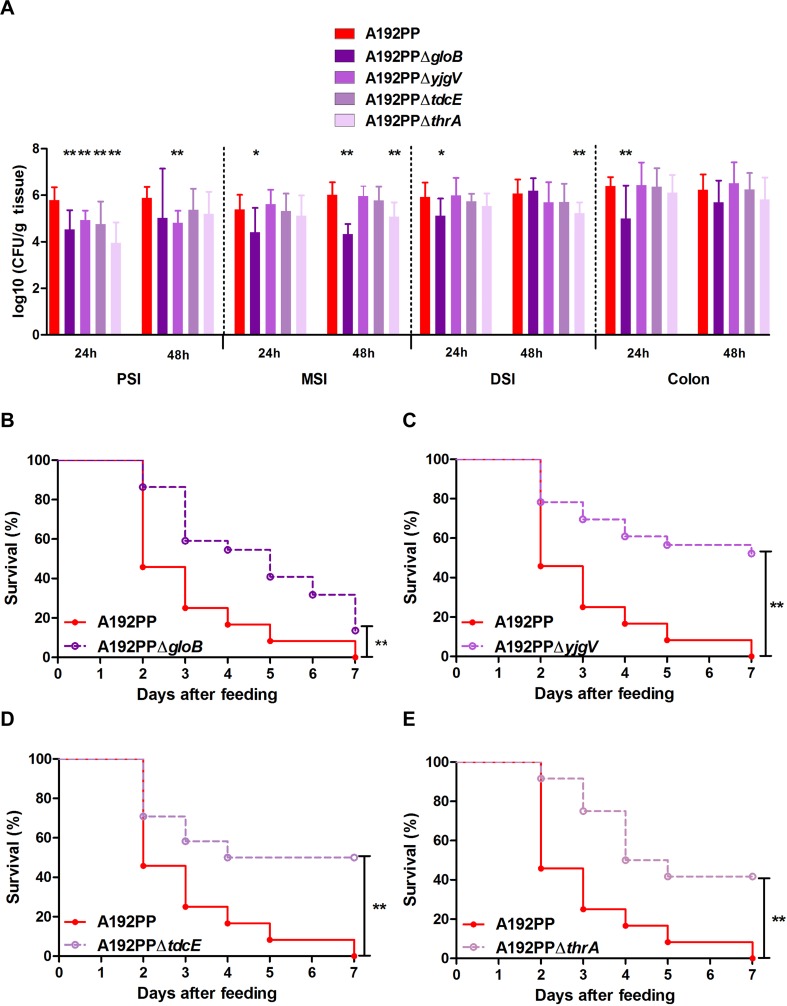
Pathoadaptive mutations of *E*. *coli* A192PP impact on GI colonization and virulence in the P2 rat. (A) Quantification of viable *E*. *coli* K1 in the gut; PSI, MSI and DSI: proximal, mid- and distal sections of the small intestine. Tissues were removed 24 or 48 h after initiation of colonization by oral administration of 2–6 x 10^6^ *E*. *coli* A192PP and CFU/g tissue determined by viable counting. *E*. *coli* K1 colonies were detected using K1-specific phage. Student's *t* test; *, *P* < 0.05; **, *P* < 0.01. (B-E) Survival of pups fed 2–6 x 10^6^ *E*. *coli* A192PP or A192PP deletion mutants at P2: *n* = 24 for each strain in each experiment. Log-rank (Mantel-Cox) test; ns, non-significant; *, *P* < 0.05; **, *P* < 0.01.

The SNPs acquired by *E*. *coli* A192PP after passage were not located in genes encoding known virulence factors or their regulators, and the molecular information available did not provide clear evidence for or against the involvement of each SNP in altered molecular function, bacterial phenotype or cellular function. No mechanisms which could determine the individual role of each gene in the enhanced virulence of *E*. *coli* A192PP strain were apparent and we believe it unlikely that undetected gross genetic rearrangements would account for the altered phenotype. We therefore compared phenotypic characteristics of wildtype and mutant *E*. *coli* A192PP isogenic strains. *E*. *coli* A192PPΔ*gloB*, A192PPΔ*yjgV*, A192PPΔ*tdcE* and A192PPΔ*thrA* were constructed by λ Red recombineering and deletions confirmed by whole genome sequencing; individual mutants had not acquired additional mutations in characterized virulence factors or larger genomic insertions, deletions, rearrangements or duplications. None of the mutants grew more slowly than the parental strain in nutrient-replete growth medium (Fig 3B). All four mutants displayed small changes to the whole-cell proteome; spots modulated at least twofold represented proteins associated with metabolism and protein synthesis (Fig 3C). Previous studies have indicated that genetic changes enhancing metabolic efficiency also enhance colonization; a mutant of *E*. *coli* K12 selected by passage through the GI tract of streptomycin-treated mice had colonization traits superior to the parent and grew more rapidly on a range of carbon sources in minimal medium than the wild type [44,45]. To determine if deletion of the four genes implicated in virulence/colonization enhancement reduced the capacity of *E*. *coli* A192PP to produce lethal infection after GI colonization, each mutant was used to colonize P2 neonatal rat litters. With all four mutants, there was decreased capacity to colonize the PSI; this was particularly evident 24 h after initiation of colonization (Fig 4A). *E*. *coli* A192PPΔ*gloB* was also significantly compromised in its capacity to colonize the MSI, DSI and colon compared to the parent strain. All four mutants displayed a significantly reduced lethal effect (Fig 4B); numerous attempts were made to complement the mutations with the corresponding functional gene by plasmid transformation as described [21] but in all instances plasmid-borne genes were rapidly lost *in vivo*. Deletions were confirmed by whole-genome sequencing of the four mutants.

As the evidence provided in Fig 4 suggests that the enhanced capacity of *E*. *coli* A192PP to cause lethal infection may be due to quantitatively superior colonization dynamics, we examined the growth characteristics of the A192 series in minimal medium supplemented with different sources of carbon (Table 2). In contrast to earlier studies [44,45], we did not find a clear correlation between GI colonizing capacity and growth rate on a range of carbon sources. *E*. *coli* A192PP grew significantly more slowly in M9 medium supplemented with D-mannose, D-galactose and succinic acid than *E*. *coli* A192. Conversely, the derivative appeared to grow more rapidly with D-ribose, N-acetylglucosamine and D-fucose as sole source of carbon but values did not reach levels of significance. These traits were not evident with *E*. *coli* A192P. Most surprisingly and unlike *E*. *coli* A192 and A192P, A192PP did not replicate over the 15 h incubation period with glucose as carbon source.

**Table 2:** Growth of *E*. *coli* K1 strains in minimal medium containing a single carbon source.

Carbon Source (0.2%)	Mean Generation Time (min) ±SD (*n* = 3)		
	A192	A192P	A192PP
D-glucose	72 ±2.27	65 ±6.24	No growth
D-mannose	113 ±2.54	107±3.68	127±1.67 **
D-galactose	65 ±2.10	68 ±1.46	77 ±4.66 **
Succinic Acid	80 ±6.63	79 ±19.93	99 ±3.95 **
D-ribose	105 ±2.35	90 ±4.97 *	99 ±8.13
N-acetylglucosamine	86 ±9.15	68 ±4.67 *	80 ±7.47
D-fucose	76 ±4.17	71 ±4.80	71 ±1.40

* *P* < 0.05

** *P* < 0.01

## Discussion

Clones of *E*. *coli* K1 are responsible for severe systemic infections in the human neonate due to their capacity to stably colonize the neonatal GI tract and overcome a number of formidable physiological barriers that, when fully formed, prevent access of these pathogens to the blood compartment. Thus, the colonizing bacteria have a restricted window of opportunity to translocate from the gut lumen to the systemic circulation which can be replicated in neonatal rodents, facilitating meaningful investigation of the nature of this age-dependency in experimental animal models [12,13]. Many *E*. *coli* K1 clinical isolates colonize the GI tract of the neonatal rat with efficiency at or approaching 100%. However, even very effective colonizers show a variable capacity to invade the blood circulation or gain access to vital organs such as the brain, with the most virulent eliciting bacteremia in less than 50% of colonized rats; induction of lethality by almost all clinical isolates occurs with a considerably lower incidence in this model of infection [12]. This redundancy has made it difficult to investigate host–pathogen interactions, with relatively large numbers of animals required to achieve levels of statistical significance for any given trait. In order to circumvent this problem and reduce animal wastage, we increased the virulence of an efficient colonizer, *E*. *coli* A192, by two rounds of passage through susceptible neonatal rat pups [17,19]. The passaged strain, *E*. *coli* A192PP, elicits bacteremia and lethality in all colonized susceptible pups and has enabled wide-ranging studies of the molecular pathogenesis of *E*. *coli* K1 experimental systemic infection [16]. We have now undertaken comparative *in vitro* and *in vivo* studies of *E*. *coli* A192 and derivatives to shed light on the dynamics of virulence enhancement.

The initial evaluation of the capacity of *E*. *coli* K1 isolate A192 to cause bacteremia and lethality after GI colonization was undertaken in the early 1980s [12,15]. In this study, Achtman and co-workers colonized five- to seven-day-old (P5-P7) Wistar rat pups and recorded an incidence for bacteremia and lethality in a minority of animals (35% and 25% respectively); these parameters were determined three or four days after colonization by oral feeding of bacterial culture. We examined this isolate some twenty years later in younger (P2) Wistar pups and found bacteremia in 23% of colonized pups over a six day observation period. In the re-evaluation reported here, we found a higher degree of lethality in comparison to our initial report, although *E*. *coli* A192 remained significantly less virulent than passaged *E*. *coli* A192PP when evaluated by the Mantel-Cox log-rank test (Fig 1). Survival was highly reproducible when the procedure was repeated using different batches of animals from the same supplier. As noted above, there were differences in experimental design between our studies and those of Achtman that account for differences in susceptibility to infection, in particular the age at which the pups were colonized. Identical procedures were used in the current study and in our earlier report and we surmise that the continuous commercial breeding programme resulted in small but significant changes in the susceptibility of the neonates to infection over the intervening period, in addition to the impact of factors beyond our control.

*E*. *coli* A192 was found to be a member of the ST95 group of K1 isolates and clustered with a number of more recent UK and USA isolates. Surprisingly, very few mutations were required to extend its capacity for systemic invasion to all colonized P2 pups. The intermediate derivative strain *E*. *coli* A192P accumulated three SNPs in genes encoding metabolic enzymes and possessed comparable virulence to *E*. *coli* A192PP (Fig 1A); a further SNP in the promoter for *thrA*, encoding enzymes involved in the conversion of L-aspartate to L-threonine [43], was found in *E*. *coli* A192PP. Thus, the four mutations are in genes or regulatory elements involved in carbon and energy metabolism and no genes encoding virulence factors or other cell structures more directly associated with pathogenesis were mutated. There is strong evidence that translocation of *E*. *coli* K1 strains from the gut lumen to the blood *via* the lymphatic system occurs with low frequency and high numbers of these bacteria in the GI tract are required to engender systemic invasion [12,46]. The numbers of colonizing *E*. *coli* K1 in each of the regions of the small intestine sampled were greater for the passaged strains than for *E*. *coli* A192 when sampled 24 h after initiation of colonization (Fig 1B), suggesting that their increased invasive capacity was related to the higher populations that were attained in the small intestine. Interestingly, although the invasive potential of *E*. *coli* A192P and A192PP was comparable, populations of the twice-passaged derivative were higher.

These data bear comparisons to earlier studies of the relationship between metabolic activity, specifically utilization of carbon sources, and the GI colonization potential of *E*. *coli* K12 [44,45]. These studies showed that generation of deletions within *flhD* (encoding the flagellar transcriptional regulator), either by GI passage or allele replacement, led to elevated expression of genes involved in carbon and energy metabolism, more efficient carbon source utilization and a higher intestinal colonizing population. We have also noted the link between modulation of metabolic capacity and the size of the colonizing *E*. *coli* population but find a more complex picture in relation to the metabolic changes that accompany increases in colonization. The previous studies found that the high colonizers were, without exception, able to grow faster on each of 11 sources of carbon and energy in minimal medium [44]; differences were attributable solely to *flhD* deletion, as whole-genome sequencing revealed no changes to the genome other than the *flhD* deletion [45]. In contrast, we found both enhanced and suppressed utilization of carbon sources by *E*. *coli* A192PP (Table 2), possibly reflecting variable impacts of each SNP on metabolic integrity. In particular, unlike both the parent and *E*. *coli* A192P, the twice-passaged strain was unable to use D-glucose for growth. Disruption of pyruvate formate-lyase activity, such as that represented by the *tdcE*-encoded 2-keto acid formate-lyase, by the SNP may be responsible for this deficit [41] but as *E*. *coli* A192P also contains this mutation and grows rapidly on glucose substrate it is hard to explain without invoking augmentation of metabolic impairment by the subsequent *thrA* promoter mutation.

TdcE is encoded within the polycistronic *tdcABCDEFG* operon that facilitates the anaerobic conversion of L-threonine to propionate [40]. Both *gloB* and *thrA* gene products may also impact on metabolism of L-threonine. Glyoxalase II, encoded by *gloB*, is a component of the glutathione-dependent methylglyoxal degradation pathway; this secondary metabolite is a toxic intermediate generated by metabolism of L-threonine and other amino acids [37,47]. As previously noted, two steps in the conversion of L-aspartate to L-threonine are catalysed by ThrA [43]. However, any relationships between enhanced colonization, invasive capacity and metabolism of L-threonine are presently unclear, particularly as we have no understanding of the impact of the SNPs on the *E*.*coli* A192 phenotype other than in relation to the increased colonizing capacity and infectivity reported here. The remaining SNP appeared in the *yjgV* gene, one of a small number of genes encoding enzymes of the Entner-Doudoroff (ED) pathway inducible by gluconate [38,48]. The capacity of *E*. *coli* K12 and the human commensal strain *E*. *coli* F-18 to colonize the GI tract of streptomycin-treated mice, abrogated by insertional deletion of *eda*, was restored by introduction of gluconate-inducible *edd* [20]; both genes encode enzymes of the ED pathway [48] and this data implicates gluconate utilization and the ED pathway as important elements of GI tract colonization by *E*. *coli*. Deletion of *yjgV* in *E*. *coli* A192PP reduces the number of colonizing bacteria in the small intestine (Fig 4A) and has a large impact on the lethality of this strain (Fig 4C), further supporting a role for gluconate metabolism in GI tract colonization by *E*. *coli*.

Overall, our data strongly suggest that the increase in invasive capacity of *E*. *coli* A192 following two rounds of passage through a host susceptible to systemic infection is due to small changes in the sequence of a limited number of metabolic genes that lead to a ten- to one-hundredfold increase in the numbers of colonizing bacteria. Deletion of any of the genes acquiring SNPs during passage resulted in reduced colonizing capacity and lethality (Fig 4). The critical threshold concentration of K1 bacteria in the small intestine for translocation from gut to blood is in the region of 10^4^−10^6^ CFU/g tissue (Fig 4B) and the integrity of the epithelial barrier in this region of the gut appears to be the key determinant of invasive infection in the neonate.
